# 1438. Prevalence and Risk Factors for Extended Spectrum Betalactamases Among Hospitalized Patients with Community Acquired Pyelonephritis in Colombia

**DOI:** 10.1093/ofid/ofab466.1630

**Published:** 2021-12-04

**Authors:** Mariana Franco Rodríguez, Jorge Cortes

**Affiliations:** 1 Universidad Nacional de Colombia- Hospital Universitario Nacional, Bogotá, Cundinamarca, Colombia; 2 Universidad Nacional de Colombia, Bogota, Distrito Capital de Bogota, Colombia

## Abstract

**Background:**

Urinary tract infections (UTI) are the most frequent bacterial infection in hospitalized patients. Extented spectrum betalactamases (ESBL) producing bacteria causing UTI have become more prevalent. Escherichia coli (E. coli) is the most frequent ESBL producing bacteria isolated in UTI. This drug resistant organisms are associated with poorer outcomes for patients. In low income countries, approaching to and treating ESBL E. coli, represent a major challenge for health care centers.

**Methods:**

A retrospective cohort of adult patients with community acquired pyelonephritis caused by Escherichia coli was identified in a tertiary hospital in Colombia. Susceptibility was performed with Vitek (BioMerieux, France); extended spectrum beta lactamase (ESBL) production was defined phenotypically. Inclusion criteria were adult patients hospitalized with a positive urine culture for E. coli. Demographic and clinical characteristics were searched in electronic records. Risk factors associated with ESBL production were identified by using a multivariate logistic regression analysis.

**Results:**

During 7 years 817 patients with pyelonephritis caused by E. coli were identified. 79 (9.7%) of them were caused by ESBL producers. Women were 66% and 408 (74.8% of them) had menopause. Mean age was 64.2 years (standard deviation of 19.1). Of the cohort, 481 (561.1%) had at least some comorbidity and was frequent to find diabetes (18.5%), immunosuppression due to oncologic disease or medications (18.4%), urolithiasis or previous surgical procedures (17%). After logistic regression, risk factors identified to predict ESBL production, were: being a man (aOR 5.4, 2.1-18.2), a woman with menopause (aOR 2.9, 1.3 -9.9), and the Charlson score (aOR 0.83, 0.73 – 0.96). Previous antibiotic use was not related to ESBL infection.

**Conclusion:**

In this relatively large cohort of patients with pyelonephritis caused by E. coli, ESBL production risk factors were not clearly identified other than sex and menopause. Curiously, Charlson score predicted a lower risk of resistance. Other factors (food consumptions and others) might be driving the resistance in the community in E. coli.

**Disclosures:**

**Jorge Cortes, MD**, **Pfizer** (Research Grant or Support)

